# Soil bacterial community characteristics and its effect on organic carbon under different fertilization treatments

**DOI:** 10.3389/fmicb.2024.1356171

**Published:** 2024-03-27

**Authors:** Chenchen Kong, Shiwen Zhang, Shengjun Yuan, Weirui Wang, Xiaoxin Song, Dandan Guo, Abubakar Sadiq Lawi

**Affiliations:** ^1^School of Earth and Environment, Anhui University of Science and Technology, Huainan, China; ^2^Miyun District Soil and Fertilizer Workstation, Beijing, China; ^3^Beijing Cultivated Land Construction and Protection Center, Beijing, China

**Keywords:** different fertilization treatments, bacterial community, soil organic carbon, metabolic functions, impact mechanism

## Abstract

**Introduction:**

By implementing small-scale and efficient fertilization techniques, it is possible to enhance the activity of microorganisms, thereby improving soil carbon sequestration and ecological value in agriculture.

**Methods:**

In this study, field experiments were conducted using various types of fertilizers: organic fertilizer, microbial fungal fertilizer, composite fertilizer, and an unfertilized control (CK). Additionally, different dosages of compound fertilizers were applied, including 0.5 times compound fertilizers, constant compound fertilizers, 1.5 times compound fertilizers and CK. Using advanced technologies such as Illumina MiSeq high-throughput sequencing, PICRUSt2 prediction, Anosim analysis, redundancy analysis, canonical correlation analysis, and correlation matrix, soil organic carbon (SOC) content and components, bacterial diversity, metabolic functions, and interaction mechanisms were examined in different fields.

**Results and Discussion:**

The results showed pronounced effects of various fertilization modes on SOC and the bacterial community, particularly in the topsoil layer (0–20 cm). Organic fertilizer treatments increased the richness and diversity of bacterial communities in the soil. However, conventional doses and excessive application of compound fertilizers reduced the diversity of soil bacterial communities and SOC content. Additionally, different fertilization treatments led to an increase in easily oxidizable organic carbon (EOC) contents. Interestingly, the relationship between SOC components and soil bacteria exhibited inconsistency. EOC was positively correlated with the bacterial diversity index. Additionally, Chloroflexi exhibited a negative correlation with both SOC and its components. The influence of metabolismon primary metabolic functions on the content of SOC components in the soil was more notable. It included seven types of tertiary functional metabolic pathways significantly correlated with SOC components (*p* < 0.05).

**Purpose and Significance:**

These findings enhance the understanding of the relative abundance of bacterial communities, particularly those related to the carbon cycle, by adjusting agricultural fertilization patterns. This adjustment serves as a reference for enhancing carbon sinks and reducing emissions in agricultural soils.

## 1 Introduction

The carbon pool of agricultural ecosystems forms an integral part of the carbon pool of terrestrial ecosystems. The carbon dioxide (CO_2_) released from soil respiration can influence the carbon balance of ecosystems, identifying carbon sequestration in agricultural soils as a critical way to reduce CO_2_ emissions (Zhang et al., 2020; Han et al., 2021; Liu et al., 2022). The active component of soil organic carbon (SOC) can reflect short-term changes in soil quality and plays a crucial role in farmland soil material cycling (Luo et al., 2022). Different field management practices are essentially ways to influence carbon sequestration in agricultural soils. Among these various practices, fertilizer application is the most common field management practice applied to maintain the yield and productivity of agricultural land. Fertilizer application can substantially influence the physicochemical properties of continuously cultivated farmland soils, and the effects of different fertilizer types and inputs on SOC vary to a certain extent. Between 1980 and 2014, nitrogen fertilizer consumption in China increased nearly 2-fold (Liu et al., 2016). It has been shown that the long-term irrational use of large quantities of chemical fertilizers caused a decrease in the carbon pool of China's agricultural soils, an imbalance in the structure of bacterial communities, and degradation of soil fertility (E et al., 2018). Since 2015, China has vigorously promoted numerous measures to optimize agricultural fertilization in various areas of the country. This initiative has positively affected the carbon sink capacity of agricultural soils (Liu et al., 2023). In recent years, the theory of the “soil microbial carbon pump” has been proposed. This theory indicates that microorganisms are essential contributors to the stabilization of the SOC pool, as they transform and regulate the formation of SOC (Cotrufo et al., 2015; Liang et al., 2017). Moreover, as the nitrogenous compounds of bacterial cell walls are mainly composed of easily decomposable peptidoglycan (Domeignoz-Horta et al., 2021; Bonner et al., 2022), they are more advantageous for soil nutrient turnover. Research has shown that organic carbon utilization by bacteria is more inclined to active components (Yang et al., 2022). Their communities and functions are susceptible to environmental control, and respond strongly to fertilizer application and other farmland management practices. When the soil environment changes, bacteria can respond rapidly, thus affecting both the soil organic matter transformation and carbon cycle (Jobbágy and Jackson, 2000; Mahoney et al., 2017). Therefore, examining the variability of organic carbon levels and bacterial communities in soils under different fertilization methods and their response relationships is of practical importance for carbon sequestration and the sustainable development of agricultural soils.

Currently, the most common types of fertilizers used in China's agricultural production are chemical fertilizers, organic fertilizers, and straw return to the field. Additionally, microbial agents are also applied. Many studies have shown that applying organic fertilizers and returning straw to the field can effectively improve the organic carbon pool in the soil. As shown by relevant experimental studies conducted in Europe, returning straw to the field can increase the SOC content by 5–50% (Gattinger et al., 2012; Maillard and Angers, 2013). Other studies showed that long-term fertilization with chemical fertilizers significantly increased the bacterial diversity and the SOC content in the soil (Dong, 2008; Guo et al., 2016). According to further research, soils under organic fertilization did not show a significant change in the organic carbon content compared to soils under chemical fertilization (Leifeld et al., 2009). The variability of organic carbon levels and bacterial communities in soils under different fertilization treatments and the mechanism underlying their effects still remain unclear. To improve the carbon sequestration capacity of agricultural soils, research needs to focus on the impact of fertilizer inputs on the changes in the amount of organic carbon in the soil. Moreover, the changes in the soil bacterial community and its response relationship to SOC after fertilization need to be clarified. In turn, it is possible to adjust the fertilization pattern to change the bacterial community structure, increase the relative abundance of bacterial communities with carbon cycle-related function, and promote SOC sequestration. This study analyzes the change characteristics of soil bacterial diversity and the metabolic function, as well as SOC and its component content of surface and sub-surface soils (0–40 cm depth) with different fertilization treatments through field experiments. The relationship between soil bacterial community and metabolic function on organic carbon is established. The aim of this study was to enhance the bacterial functional activity and soil carbon sequestration capacity through efficient fertilization approaches based on long-term positioning experiments. This study is of practical significance for improving the ecological value and carbon sink function of farmland soils.

## 2 Materials and methods

### 2.1 Experimental area and treatment design

To examine the effects of different fertilizer application methods on soil bacterial communities, metabolic functions, SOC levels, and active SOC components, field experiments were conducted in 2022 at the JXY Base of Beijing Miyun District, China (40°17′47″ N and 116°47′10″ E). The climate in this area is a warm temperate, semi-humid, semi-arid monsoon climate, with an annual average temperature of 10.8°C. The experimental soil type is brown soil with sandy loam and alluvial parent material. The primary cultivation type is summer maize–winter wheat crop rotation. Prior to the experiment, the soil had an organic matter content of 12 g·kg^−1^, a total nitrogen (TN) content of 0.71 g·kg^−1^, and a water content of 15.96%.

**Figure 1:**
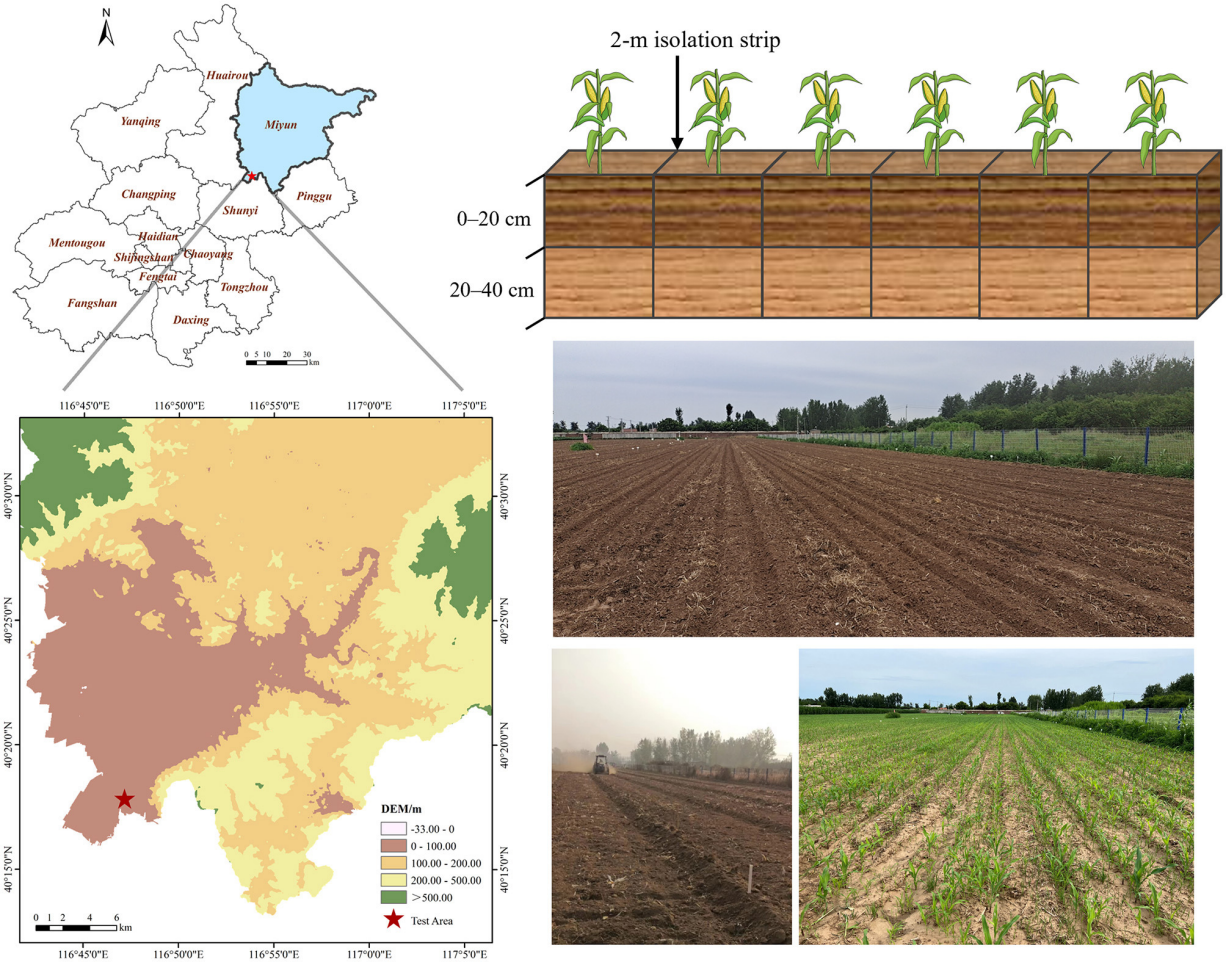
Overview of the experimental area.

For the field experiment, this study used different fertilizer application types and dosages as experimental design variables based on the conventional fertilization mode of agricultural production. The experiment included a total of six treatments, each of which was replicated three times, totaling 18 experimental areas. Each area had a size of 84 m^2^ (14 × 6 m), and areas were arranged randomly. A 2-m isolation strip was left around each treatment area to avoid mutual interference between treatments. After the wheat harvest of the previous season, rotary tillage returned both above ground and rooted straw directly into the soil. The amount of fertilizer to be applied was based on the native fertility of the soil, the crop yield, and the nutrient ratio of the applied fertilizer. Both organic and compound fertilizer applications were measured in nitrogen (N), using the conventional application dose of 194.40 kg·ha^−1^. Table 1 and Figure 1 provide the details of different experimental treatment settings as well as an overview of the experimental area. As compound fertilizer, Root Lido organic-inorganic compound fertilizer was used (N: P_2_O_5_: K_2_O = 22:10:10, organic matter ≥ 15%, effective live bacteria ≥ 0.2 billion·g^−1^). As organic fertilizer, Beijing Woshengjie commercial organic fertilizer was used (organic matter ≥ 45%, N ≥ 1.5%, moisture content ≤ 30%). As microbial bacterial agent, magic grain live bacteria in 13 trace element water-soluble fertilizers were used. All other farm management practices during planting were consistent with local maize production practices. All fertilizers were applied only once as a base fertilizer one day before corn planting, with no follow-up fertilization. A common variety of seed maize (Dongdan 6531) was used for the experiment, which was sown on June 25, 2022, and harvested on October 9, 2022.

**Table 1:** Details of different experimental treatment settings.

**Different treatments**	**Rate of fertilizer application/kg**	**Fertilizer details**
OF	567.00	Beijing Woshengjie commercial organic fertilizer (organic matter ≥ 45%, N ≥ 1.5%, moisture content ≤ 30%)
MF	0.19	Magic grain live bacteria in 13 trace element water-soluble fertilizer
CFa	19.28	Root Lido organic-inorganic compound fertilizer (N: P_2_O_5_: K_2_O = 22:10:10, organic matter ≥ 15%, effective live Rbacteria ≥ 0.2 billion·g^−1^)
CF	38.56	
CFb	57.83	
CK	—	—

In the different treatments, OF, MF, CFa, CF, CFb, and CK corresponded to the application of organic fertilizer under straw-returning condition, microbial fungicide under straw-returning condition, 0.5 times of compound fertilizer under straw-returning condition, constant compound fertilizer under straw-returning condition, 1.5 times of compound fertilizer under straw-returning condition, and no fertilizer under straw-returning condition, respectively. Same as below.

### 2.2 Sampling and analysis

#### 2.2.1 Sample collection

Soil samples were collected before the corn harvest in October 2022. Samples were collected from the 0–20-cm and 20–40-cm soil layers between maize plants (away from crop roots) in each experimental plot, using a soil drill with a diameter of 5 cm. The five-point method was applied for sampling, and samples of the same treatment were mixed to obtain one composite sample per site. Three composite samples were collected per treatment. Soil samples were divided into three parts after sieving to remove impurities. One part was stored at −80°C for 16S rRNA sequencing, one part was stored at 4°C and refrigerated for dissolved organic carbon (DOC) determination, and one part was naturally dried and sieved to determine other organic carbon fractions and the chemical properties of the soil.

#### 2.2.2 DNA extraction, sequencing, and species annotation of soil bacteria

Total genomic DNA was extracted from soil samples using the QIAGEN DNeasy Power Soil Kit (Kaijie, Shanghai, China) kit and DNA concentration was detected using a Qubit 3.0 Fluorometer (Life Technologies, Carlsbad, CA, USA). The integrity of the extracted genomic DNA was measured by 1% agarose gel electrophoresis. Amplification of the V3–V4 region of the 16S rRNA gene by PCR was performed using the forward primer 341F (5′-CCTACGGGNGGCWGCAG-3′) and the reverse primer 805R (5′-GACTACHVGGGTATCTAATCC-3). After PCR amplification, products were recovered by passivation with magnetic beads. Qubit was used to measure the product concentration, and the band size of PCR products was obtained via 1.5% agarose gel electrophoresis. The products recovered by PCR amplification were mixed after fluorescence quantification. According to the quantification results, each sample was mixed in the corresponding proportion according to the sequencing volume requirement of each sample. Sequencing libraries were prepared using the TruSeq Nano DNA LT Library Prep Kit from Illumina. These DNA libraries were quality-checked using an Agilent analyzer 2100 system, tested, and sequenced using the Illumina Miseq amplicon sequencing platform with a PE250 sequencing strategy. The mixed sample library was barcode identified, and low-quality filtering was performed using Trimmomatic v. 0.39 software: the window size was set to 50 bp, and the average quality value within the window exceeded 20. After this, the primer sequences were identified and removed by Cutadapt v. 3.5 software to obtain clean sequences without primers. The amplicon sequencing variant table was obtained after quality filtering, denoising, merging, and removing chimeras from sequences using DADA2. Low-abundance amplicon sequencing variants were filtered using QIIME2 software (v. 2022.3). Silva (release 138) was selected as reference database, and the Naive Bayes species classifier was constructed using QIIME2 to annotate species with the feature sequences of each amplicon sequencing variant (Martin, 2011; Bolger et al., 2014; Callahan et al., 2016).

#### 2.2.3 Determining organic carbon and chemical properties of soils

SOC was determined using the potassium dichromate external heating method, DOC was determined using the K_2_SO_4_ leaching method, and easily oxidizable organic carbon (EOC) was determined using the UV spectrophotometric method. Other chemical properties of soil samples were tested according to Soil Agrochemical Analysis (3rd edition) (Bao, 2000). Soil organic matter (SOM) was measured via the potassium dichromate volumetric method with external heating. TN was determined by the Kjeldahl method. Available phosphorus (AP) was measured via the 0.5 mol·L^−1^ NaHCO_3_ leaching and molybdenum antimony colorimetric method. Available potassium (AK) was determined via the 1 mol·L^−1^ NH_4_OAc extraction and flame photometric method. The soil pH was measured using a pH meter (JC-18007), and electrical conductivity was determined using a conductivity meter (JC-17005).

#### 2.2.4 Data processing and analysis

PICRUSt2 software was used to predict the functions of soil bacteria, based on KEGG comparative annotation, to analyze the metabolic pathways of gene products in experimental plots and their functions. IBM SPSS 27 software was used for statistical testing of the data using one-way analysis of variance and multiple comparisons (Duncan's test) for soil bacterial α-diversity indices, and correlation analysis between SOC and bacterial communities. R v. 4.2.2 was used for Anosim analysis based on a weighted unifrac algorithm, centroid clustering analysis of bacterial metabolic functions, and canonical correspondence analysis between SOC and first-level metabolic functions of soil bacteria. Redundancy analysis between SOC, bacterial communities, and soil properties was performed using Canoco 5 software, and all plots were generated using R 4.2.2.

## 3 Results and analysis

### 3.1 Characteristics of soil bacterial community diversity and metabolic function under different fertilization treatments

#### 3.1.1 Diversity of soil bacterial communities under different fertilization treatments

**Figure 2:**
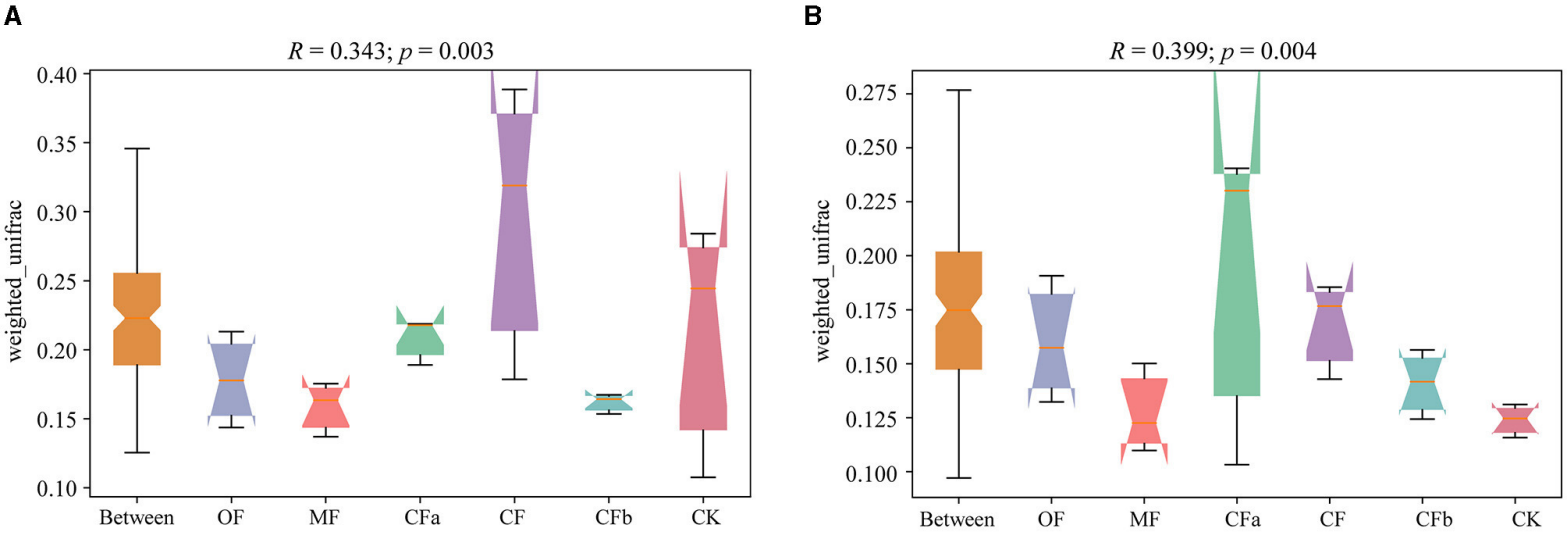
Anosim analysis of weighted unifrac algorithms. **(A)** 0–20 cm. **(B)** 20–40 cm.

Differences in soil bacterial community structures under different fertilization treatments are depicted in Figure 2. According to Anosim analysis, the *R*-values were 0.343 (0–20 cm soil depth) and 0.399 (20–40 cm soil depth), which were >0. This result indicates that intergroup differences in bacterial community structure in each soil layer under different fertilization treatments were greater than intragroup differences under the same fertilization treatment. Significant difference test showed that the *p*-values of varying soil depths were 0.003 (0–20 cm soil depth) and 0.004 (20–40 cm soil depth) of different fertilization, reaching the significance level (*p* < 0.05). Various fertilizer applications significantly changed the soil bacterial community structure.

Differences in soil bacterial community richness and diversity among different fertilization treatments were reflected by the indices of ACE, Chao1, Shannon, and Simpson. As shown in Table 2, these values were higher under the organic fertilizer treatment at different soil depths than under other treatments, but the significance level was not reached. The abundance and diversity of bacteria in the 0–20-cm soil layer gradually decreased with increasing compound fertilizer level. The abundance and diversity of soil bacteria under the CFb treatment were lower than those under CK, and different fertilizer treatments had no significant effect on ACE, Chao1, and Shannon (*p* < 0.05). The abundance and diversity of bacteria in the 20–40-cm subsurface soil layer was lowest under compound fertilizer application.

**Table 2:** Alpha diversity index values of soil bacteria under different fertilization treatments.

**Soil depth**	**Fertilization treatment**	**ACE**	**Chao1**	**Shannon**	**Simpson**
0–20 cm	OF	3,141.161 ± 871.530^a^	3,156.758 ± 885.623^a^	10.623 ± 0.441^a^	0.999 ± 0.001^a^
	MF	3,092.978 ± 445.827^a^	3,110.405 ± 451.213^a^	10.597 ± 0.183^a^	0.999 ± 0.001^a^
	CFa	3,061.132 ± 319.590^a^	3,087.913 ± 326.531^a^	10.415 ± 0.166^a^	0.998 ± 0.001^ab^
	CF	2,781.431 ± 911.732^a^	2,799.599 ± 923.203^a^	10.231 ± 0.800^a^	0.998 ± 0.001^ab^
	CFb	2,238.310 ± 575.971^a^	2,250.063 ± 580.132^a^	9.800 ± 0.520^a^	0.997 ± 0.001^b^
	CK	2,803.476 ± 841.209^a^	2,811.072 ± 866.075^a^	10.371 ± 0.603^a^	0.998 ± 0.001^ab^
20–40 cm	OF	3,759.166 ± 385.908^a^	3,773.183 ± 399.175^a^	10.971 ± 0.144^a^	0.999 ± 0.001^a^
	MF	3,378.671 ± 281.186^ab^	3,411.989 ± 271.943^ab^	10.682 ± 0.113^ab^	0.999 ± 0.001^a^
	CFa	2,654.904 ± 1068.303^b^	2,663.511 ± 1078.499^b^	10.283 ± 0.527^b^	0.999 ± 0.001^a^
	CF	2,984.085 ± 174.189^ab^	2,998.260 ± 180.224^ab^	10.545 ± 0.102^ab^	0.999 ± 0.001^a^
	CFb	3,002.594 ± 65.234^ab^	3,023.212 ± 70.692^ab^	10.422 ± 0.054^b^	0.998 ± 0.001^a^
	CK	3,303.579 ± 161.884^ab^	3,307.711 ± 163.030^ab^	10.638 ± 0.081^ab^	0.999 ± 0.001^a^

Different lowercase letters indicate a significant difference among fertilization treatments at the same soil depth (*p* < 0.05).

#### 3.1.2 Differences of soil bacterial metabolic functions under different fertilization treatments

**Figure 3:**
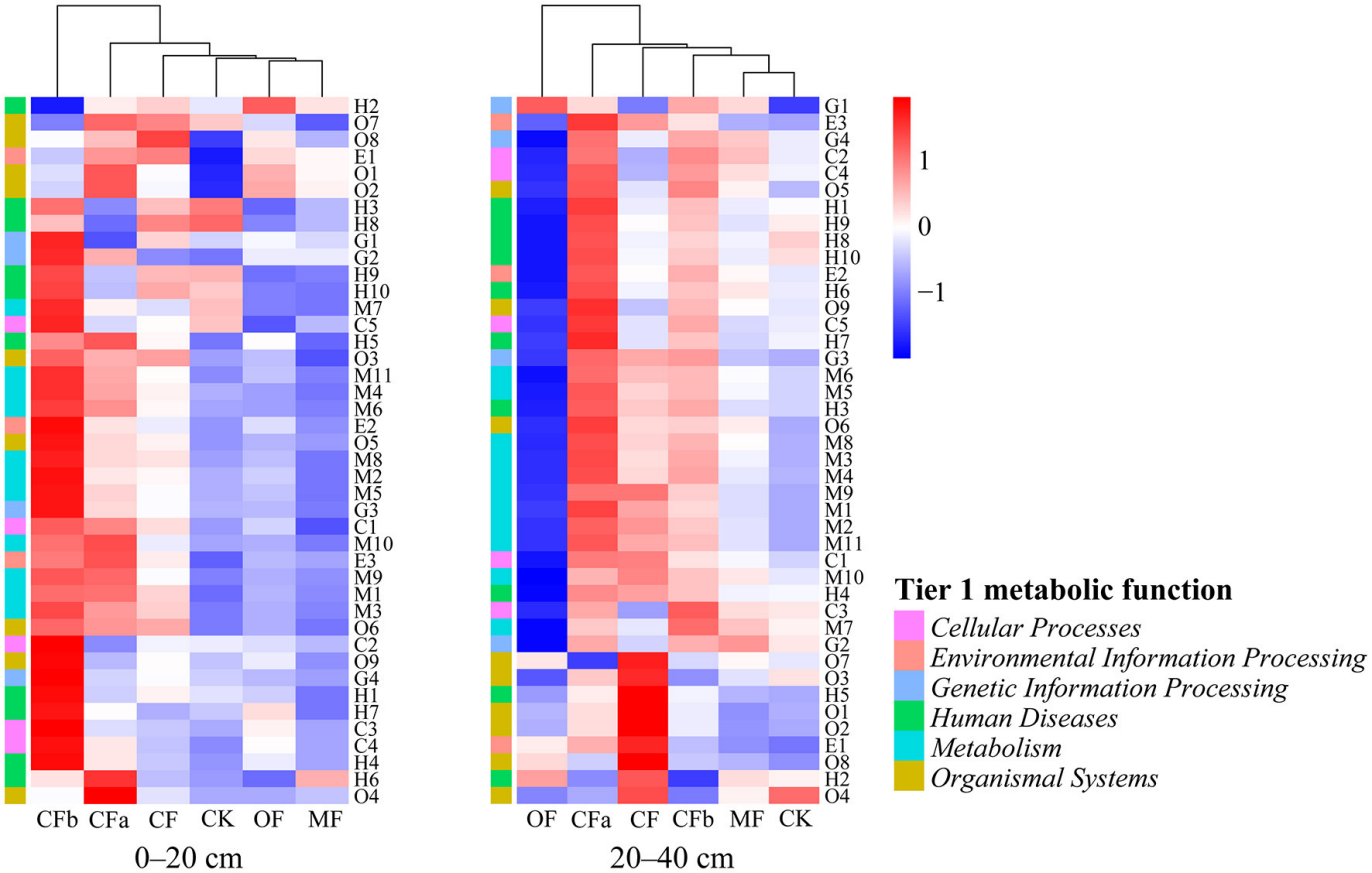
Functional characteristics of the soil bacterial metabolism under various fertilization treatments. M1–M11 correspond to Secondary metabolic functions in the order of xenobiotics biodegradation and metabolism, nucleotide metabolism, metabolism of terpenoids and polyketides, metabolism of other amino acids, metabolism of cofactors and vitamins, lipid metabolism, glycan biosynthesis and metabolism, energy metabolism, carbohydrate metabolism, biosynthesis of other secondary metabolites, amino acid metabolism; E1–E3 correspond in order to signal transduction, membrane transport, membrane transport; G1–G4 correspond in order to translation, transcription, replication and repair, folding, sorting and degradation; C1–C5 correspond in order to transport and catabolism, cellular community—prokaryotes, cellular community—eukaryotes, cell motility, cell growth and death; H1–H10 correspond in order to neurodegenerative disease, infectious disease: viral, infectious disease: parasitic, infectious disease: bacterial, immune disease, endocrine and metabolic disease, drug resistance: antimicrobial, cardiovascular disease, cancer: specific types, cancer: overview; O1–O9 correspond in order to sensory system, nervous system, immune system, excretory system, environmental adaptation, endocrine system, digestive system, development and regeneration, circulatory system.

First-level functional prediction showed that the metabolic functions of soil bacteria under different fertilization treatments could be classified into the following six categories: *metabolism, genetic information processing, environmental information processing, cellular processes, human diseases*, and *organismal systems*. Among these, *metabolism* had the highest relative abundance of 70.53% (Figure 3). Analysis of the abundance of 42 types of secondary metabolic functions as well as the differences between groups in different fertilization treatments showed that the composition of bacterial secondary functions was similar under MF, OF, and CK treatments at a soil depth of 0–20 cm. Moreover, the relative abundance of bacterial secondary metabolic functions was higher under CFa, CF, and CFb treatments with compound fertilization, which was highest in the CFb treatment with additional compound fertilizer application. At a soil depth of 20–40 cm, the MF and CK groups could be classified into one group. The relative abundances of bacterial secondary metabolic functions in CFa, CF, and CFb of compound fertilizer application treatments were still at a high level.

### 3.2 Characteristics of soil organic carbon components under different fertilization treatments

**Figure 4:**
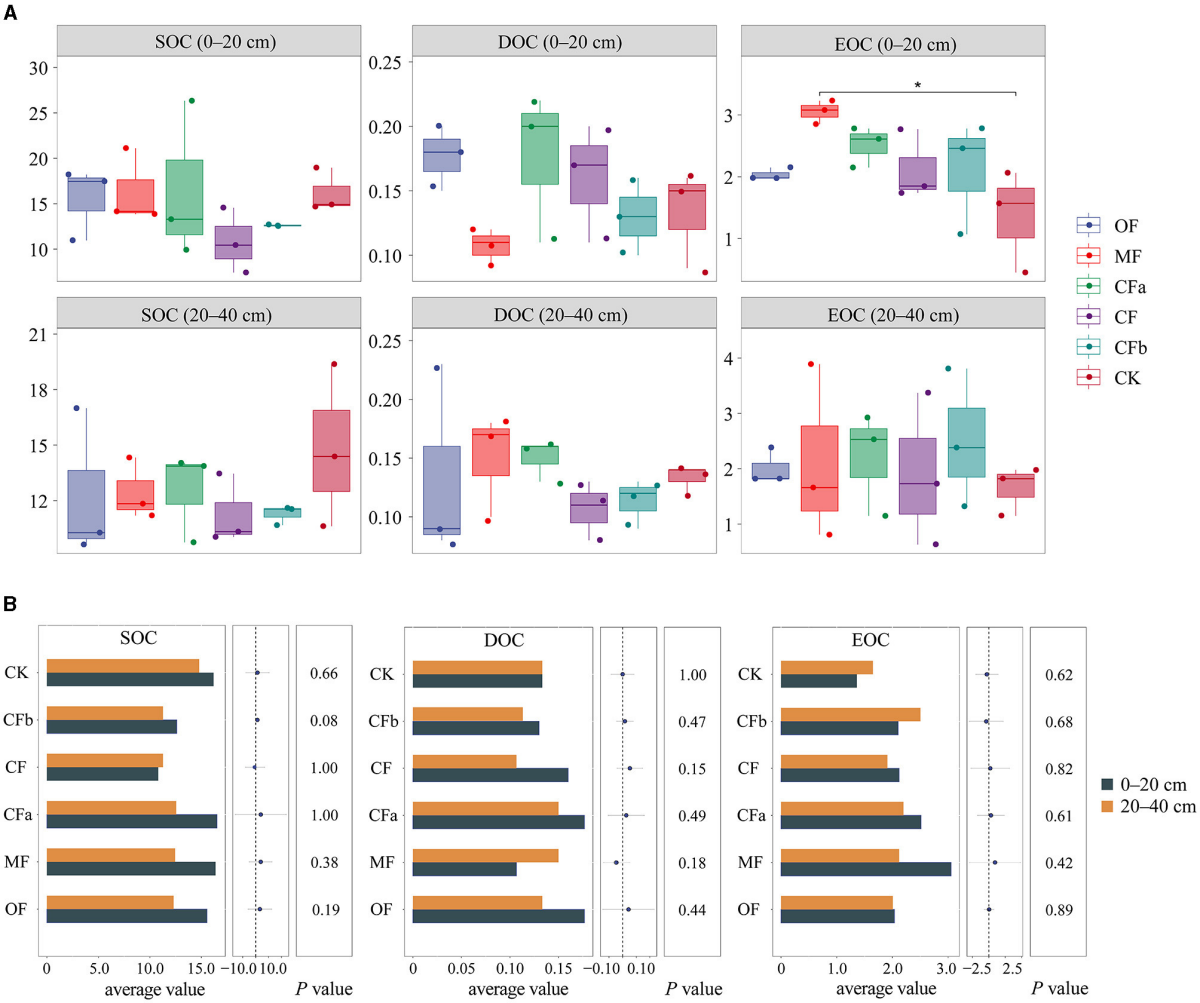
Content characteristics of soil organic carbon levels and components under different fertilization treatments. **(A)** Characteristics of soil organic carbon components under different fertilization. **(B)** Characteristics of soil organic carbon components in different depths.

As shown in Figure 4, under different fertilization treatments (OF, MF, CF, and CK), at a soil depth of 0–20 cm, both SOC and EOC contents were highest under the MF treatment, at 16.38 and 15.54 g·kg^−1^ for different fertilizer types. At a soil depth of 20–40 cm, the SOC content was highest under the CK treatment, at 14.79 g·kg^−1^, under different fertilizer types. The EOC under CK and MF treatments led to significant differences in the 0–20-cm surface soil layer (*p* < 0.05). When different doses of compound fertilizers (CFa, CF, CFb, and CK) were applied to the 0–20-cm soil layer, the contents of SOC, EOC, and DOC were highest under the CFa treatment, at 16.51, 0.18, and 2.51 g·kg^−1^, respectively. These levels eventually decreased after overdosing with compound fertilizers. The changes in SOC and DOC contents in the 20–40-cm soil layer presented the same trend as the changes of the surface soil. Overall, different fertilizer treatments increased the EOC content, which indicates a relatively high degree of activity for EOC. Conventional doses and over-application of compound fertilizers reduced the diversity of soil bacterial communities and SOC content in the 0–40-cm soil layer.

### 3.3 Relationship between bacterial communities and their metabolic functions with soil organic carbon levels

#### 3.3.1 Relationship between bacterial communities and soil organic carbon levels

**Figure 5:**
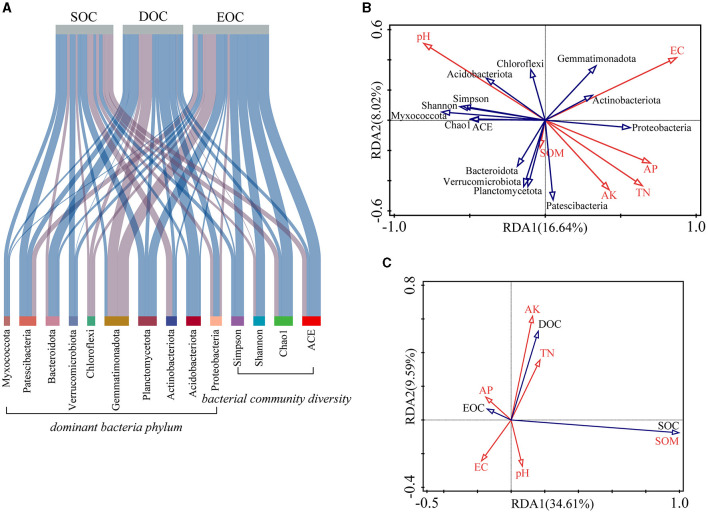
Effects of bacterial community diversity on soil organic carbon levels. **(A)** Correlation analysis of SOC with bacterial diversity and dominant bacterial phylum (the light blue line indicates a positive correlation, and the light purple line indicates a negative correlation. The thickness of the lines represents the strength of the correlation). **(B)** Redundancy analysis of bacterial community diversity and soil properties. **(C)** Redundancy analysis of SOC and soil properties.

The top 10 bacterial phyla according to their abundance levels were selected to explore the relationship between SOC components and bacterial communities in combination with bacterial diversity indices and soil properties. As shown in Figure 5A, the effects of soil bacteria on SOC and its components were inconsistent. EOC correlated positively with the bacterial diversity index, and Chloroflexi correlated negatively with both SOC and its components. The results of redundancy analysis showed that the influences of both pH and electrical conductivity on the bacterial community were highly significant (*p* < 0.01). Among them, the bacterial diversity index, Acidobacteriota and Chloroflexi was significantly positively correlated with pH while it was negatively correlated with TN, AK, and AP. Additionally, SOM positively affected Bacteroidota, Verrucomicrobiota, Planctomycetota, and Patescibacteria (Figure 5B). Each soil nutrient index (TN, AK, AP, and SOM) was positively correlated with the DOC content. Significant positive correlations were also found between AP and EOC content and between pH and SOC content (Figure 5C).

#### 3.3.2 Effects of bacterial metabolic functions on soil organic carbon levels

**Figure 6:**
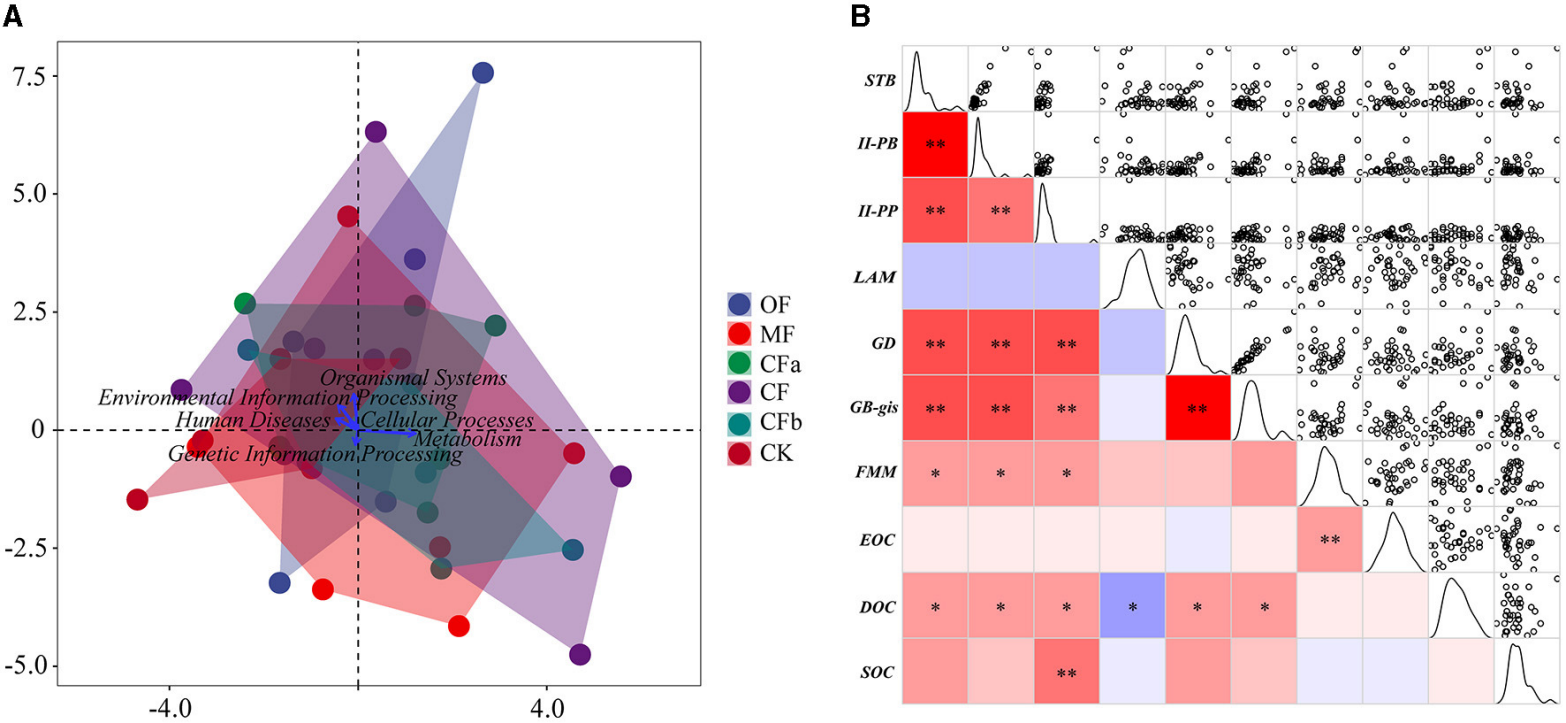
Association analysis between soil organic carbon levels and microbial metabolic functions. **(A)** Canonical correspondence analysis of primary metabolic functions and SOC components under different fertilization treatments. **(B)** Correlation matrix between soil organic carbon components and tertiary metabolic functions. The abbreviations correspond to the tertiary metabolic functions, STB, sesquiterpenoid and triterpenoid biosynthesis; II-PB, biosynthesis of type II polyketide backbone; II-PP, biosynthesis of type II potyketide products; LAM, lipoic acid metabolism; GD, glycosaminoglycan degradation; GB-gis, glycosphingolipid biosynthesis—globo and isoglobo series; FMM, fructose and mannose metabolism, respectively. The color column indicates correlation, blue indicates negative correlation, red indicates positive correlation, * indicates *p* < 0.05, ** indicates *p* < 0.0 1.

Figure 6 depicts the relationship between the metabolic functions of soil bacteria and organic carbon components. Canonical correspondence analysis (Figure 6A) of the first-level metabolic functions with SOC and its components showed that SOC dispersion was prominent among all treatment groups except for CFa and CFb. Thus, there was no specificity in the effect of metabolic functions on SOC at the first level. Among them, the *metabolism* function had the best effect on SOC content, identifying *metabolism* as an important bacterial function affecting changes in SOC. Accordingly, 134 classes of tertiary metabolic functions belonging to *metabolism* were further selected. Correlation matrix analysis showed that seven categories of functional metabolic pathways were significantly correlated with organic carbon (Figure 6B). *FMM* significantly influenced EOC, and *II-PP* was significantly correlated with SOC (*p* < 0.01). Further, DOC significantly correlated with *STB, II-PB, II-PP, LAM, GD*, and *GB-gis* (*p* < 0.05); DOC was negatively correlated with *LAM* and positively correlated with all other potential ecological functions (*p* < 0.05).

## 4 Discussion

### 4.1 Effects of different fertilization treatments on the soil bacterial community

Bacteria play a vital role in the decomposition of organic matter and the cycling of nutrients, thus enriching soils (Jin et al., 2021). The application of organic fertilizers improves the physical environment of the soil, thus ensuring that the soil can meet crop growth requirements. Using organic fertilizers alone usually results in higher nutrient input, which accelerates bacterial growth, increases bacterial activity, and enriches the bacterial community structure (Chivenge et al., 2011; Guo et al., 2019). In this study, the highest levels of bacterial diversity index values were found under the OF treatment at different soil depths. Redundancy analysis showed that SOM was positively correlated with Bacteroidota, Verrucomicrobiota, and Planctomycetota. The supply of nutrients is a critical factor in the growth of the soil bacterial community. Inputs of organic fertilizers increase the content of organic matter in soil. After its decomposition, it can be used as an essential carbon source for the growth and reproduction of microbes (Hao et al., 2019). Bacteroidota is the same nutrient-rich taxon of microorganisms as Planctomycetota, and it was found that the abundance of Verrucomicrobiota correlated positively with the content of organic matter in soil (Yu et al., 2020). Therefore, in a nutrient-rich metabolic substrate environment, the abundance of nutrient-rich taxa increased significantly. This increase indicates that the moderate application of organic fertilizers is conducive to enhancing the abundance and diversity of bacteria in soil. In contrast, the bacterial abundance and diversity of organic-inorganic compound fertilizer treatments applied in this study were relatively low and gradually decreased with increasing amount of fertilizer applied. On the one hand, the chemical compounds contained in fertilizers may raise the level of hard-to-oxidize organic materials in the soil (Zhang et al., 2009), resulting in a lack of available nutrients for bacteria. On the other hand, excessive application of compound fertilizers produces a large amount of N, thus decreasing soil pH and soil quality; this is detrimental to the growth and development of crop roots and bacterial activity, and the growth and reproduction of microbes are suppressed (Tong et al., 2009; Tian and Niu, 2015). This finding is consistent with the result that pH had a positive effect on the bacterial diversity index and an inverse effect with TN, AK, and AP. Therefore, excessive application of compound fertilizers reduced the abundance and diversity of soil bacteria. PICRUSt2 function prediction showed that the relative abundance of bacterial primary metabolic functions was the highest for *metabolism* under each fertilizer treatment at different soil depths; this result is consistent with the results of Nan et al. (2021) and Zhong et al. (2023). The primary energy sources of soil bacteria are carbohydrates and amino acids, which are also both constituents and metabolites of microorganisms. The amino acid metabolism is essential for the survival and reproduction of bacteria, as it accelerates mineralization and promotes soil nutrient cycling (He et al., 2020; Ding et al., 2021). In addition, in the present study, different fertilization treatments affected the relative abundance of each secondary metabolic function. For example, the relative abundance of secondary metabolic functions of bacteria were significantly higher under compound fertilizer application treatment. Therefore, in the future, the functional abundance of selected bacteria in soil can be directionally regulated by fertilizer application.

### 4.2 Effects of different fertilization strategies on soil organic carbon levels

As a key farm management measure, fertilizer application influences SOC accumulation. Under different fertilizer management strategies, the response degree of SOC and its components to different exogenous carbon inputs and protection mechanisms differed. This study showed that the SOC content in the topsoil layer under MF treatment was slightly higher. The reason was mainly the increased microbial activity in the upper soil layer after the application of microbial fertilizer, which stimulated dormant microorganisms. The accumulation of organic carbon in soil primarily originated from microbial activities, which accelerated the decomposition of fresh organic material (De Nobili et al., 2001; Fontaine et al., 2003). SOC and its components were also highly abundant under the CK treatment in the 0–40-cm soil layer, considering that lower N levels would reduce the effectiveness of N in the soil. As straw is a direct carbon source, the input of straw into soil increased the carbon concentration in the soil substrate. This led to nutrient limitations and forced microorganisms to mineralize more nutrient elements (such as N and P) from the SOM to ensure growth and reproduction. Because the releases of nutrients from organic carbon and organic matter in soil are coupled, nutrient release is accompanied by SOC production. However, OF treatment did not significantly increase the SOC content, which may be caused by the slow release of this fertilizer type. Therefore, organic and inorganic fertilizers are more conducive to enhancing the active SOC content (Ju et al., 2022; Lin et al., 2022). In this study, soil pH was negatively correlated with TN, EOC, and DOC. N is an essential factor influencing SOC changes (Chen et al., 2013; Song et al., 2015). The pH can also indirectly affect the input of organic carbon into the soil by changing its solubility, inhibiting the growth and activity of microorganisms, and disrupting the community structure (Lungu and Dynoodt, 2008). It has been shown that excessive fertilizer application can cause excessive N levels in soil, thus triggering a decrease in the soil pH and changes in soil properties; these changes inhibit crop root growth and microbial activity and reduce the SOC level. Substantial fertilizer application causes a decrease in the soil C/N ratio, which in turn accelerates organic carbon decomposition (Tong et al., 2009; Tian and Niu, 2015). The above-mentioned findings are consistent with the conclusion of the present study that increased application of compound fertilizers results in higher soil TN levels, lower pH levels, and lower surface SOC levels and its fractions.

### 4.3 Effects of bacteria on soil organic carbon levels and its components

Soil bacteria play an advantageous role in soil nutrient turnover owing to their unstable cell walls and ease of decomposition (Fan et al., 2021). As an essential link for the carbon cycle, bacteria mediate multiple carbon cycling processes such as carbon sequestration, carbon degradation, and methane metabolism in soils (Lehmann and Kleber, 2015); they prefer to utilize activated forms of organic carbon in this cycle (Kleber et al., 2021). The EOC was the most easily oxidized reactive organic carbon, which is extremely susceptible to microbial influences and fairly soluble. It was found to be more sensitive to the measurement of SOM than to other agriculture-related indicators. Usually, a change in carbon pool capacity occurs mainly because of the change produced by EOC (Lehmann and Kleber, 2015). This study found that EOC in soil was positively correlated with the bacterial diversity index, and the degree of association was relatively strong. This result indicates that increasing the diversity of the soil bacterial community can increase the EOC content of farmland. The representative nutritional mode of Chloroflexi is photosynthesis, where microorganisms are capable of producing energy through photosynthesis and using CO_2_ as carbon source. The members of Chloroflexi can proliferate in extreme environments and are highly competitive in soils with low nutrient contents, such as organic matter. The carbon cycle processes they participate in include CO oxidation, CH_4_ oxidation, and degradation of macromolecules such as cellulose (Hemp et al., 2015; Islam et al., 2019; Ward et al., 2019). Thus, in this study, Chloroflexi was negatively correlated with SOC and its components and nutrients, such as SOM and TN. Liang et al. (2017) suggested that SOC is heavily influenced by microbial metabolic functions. The soil microorganisms form many microbial carbon sources through a series of iterative processes such as cell generation, population growth, death, and decay, which stably exist in soils. Among the seven categories of tertiary metabolic functions that were significantly correlated with SOC and its components, *fructose and mannose metabolism* (*carbohydrate metabolism*) were positively associated with carbon and nitrogen cycling, which is consistent with the findings of Ma et al. (2021). *Lipoic acid metabolism* (*metabolism of cofactors and vitamins*) facilitates the adaptation of microbes to their environment and information transfer across the soil ecosystem (Li and Ma, 2018). The *metabolism of terpenoids and polyketides* is closely linked to the survival and development of bacterial communities (Ding et al., 2021). Therefore, the effect of bacteria on SOC must be recognized. However, the regulation of the organic carbon cycle is bidirectional. On the one hand, soil bacteria can continuously accumulate cellular residues and store them in the soil through assimilation, thus increasing SOC sequestration. On the other hand, soil bacteria can also degrade organic carbon in the soil and release CO_2_ (Camenzind et al., 2023). When the levels of exogenous carbon increase, the “stimulation effect” enhances the ability of soil microorganisms to degrade organic carbon, which leads to increased CO_2_ release. In contrast to the loss of carbon through the stimulation effect, the microbial “sequestration effect” increases biomass and residue accumulation and continues to contribute microbial carbon residues to soils. The “trade-off” between decomposition and assimilation is used to determine the organic carbon content of the soil (Zhu et al., 2020).

Based on a field experiment, this paper is only a preliminary study of the short-term changes in bacterial community diversity and function, SOC and its active components, and their relationship under different fertilizers and fertilizer dosages. Based on this preliminary experiment, the trends of organic carbon activity and stability components in soil will continually be monitored over time and the inner links with soil carbon sequestering functional microorganisms will be explored in depth in future research. The overall goal is to improve the applicability of this research for sink enhancement and emission reduction in agricultural soils and to provide a reference for the use of microorganisms to regulate soil-stabilized organic carbon levels.

## 5 Conclusions

(1) Pronounced effects of different fertilization modes on SOC and the bacterial community were found in the topsoil layer of 0–20 cm. Application of organic fertilizers increased both the abundance and diversity of bacterial communities in the soil more than application of compound fertilizers. Conventional doses and over-application of compound fertilizers reduced both the diversity of soil bacterial communities and SOC content. The degree of activity of soil EOC was relatively high, and different fertilization treatments enhanced the EOC content.

(2) EOC was found to be positively correlated with the bacterial diversity index, and Chloroflexi was negatively correlated with both SOC and its components. Changes in soil pH and EC significantly impacted the bacterial community (*p* < 0.01), and pH was positively correlated with SOC. *Metabolism* had the strongest effect on the content of SOC components in soil.

(3) The effects of soil bacteria on SOC are not uniform. Future research on efficient fertilization techniques to enhance the abundance and functional activity of bacterial communities to improve the carbon sequestration capacity of soils should consider the functional targets of different bacteria.

## Data availability statement

The data presented in the study are deposited in the NCBI Sequence Read Archive (SRA) repository, accession number PRJNA1076699. Since the data involves the key contents of the authors' doctoral dissertation, to ensure the confidentiality of the data before the publication of the doctoral dissertation, the data will be released on December 31, 2025. Requests to access the datasets should be directed to KC, cckong0123@126.com.

## Author contributions

CK: Data curation, Investigation, Methodology, Visualization, Writing – original draft, Writing – review & editing. SZ: Funding acquisition, Methodology, Writing – review & editing. SY: Resources, Writing – review & editing. WW: Resources, Writing – review & editing. XS: Investigation, Writing – review & editing. DG: Investigation, Writing – review & editing. AL: Writing – review & editing.
